# The (cost) effectiveness of guided internet-based self-help CBT for dialysis patients with symptoms of depression: study protocol of a randomised controlled trial

**DOI:** 10.1186/s12888-019-2363-5

**Published:** 2019-11-27

**Authors:** Els Nadort, Robbert W. Schouten, Friedo W. Dekker, Adriaan Honig, Patricia van Oppen, Carl E. H. Siegert

**Affiliations:** 1 Department of Nephrology, OLVG hospital, Jan Tooropstraat 164, 1061 AE Amsterdam, Netherlands; 2 Department of Psychiatry, OLVG, Jan Tooropstraat 164, 1061 AE Amsterdam, Netherlands; 3 Department of Amsterdam Public Health research institute, VUmc, Van der Boechorststraat 7, 1081 BT Amsterdam, Netherlands; 4 Department of Clinical Epidemiology, Leiden University Medical Centre, Albinusdreef 2, 2333 ZA Leiden, Netherlands; 5 Department of Psychiatry, Amsterdam University Medical Centre, Amsterdam and GGZ inGeest, Oldenaller 1, 1081 HJ Amsterdam, Netherlands

**Keywords:** Depression, Dialysis, Internet, Self-help intervention, EHealth, CBT, PST, RCT, Cost-effectiveness, Hair cortisol

## Abstract

**Background:**

Only a minority of dialysis patients with depressive symptoms are diagnosed and receive treatment. Depressive symptoms are highly prevalent in this population and are associated with adverse clinical outcomes. Underlying factors for this undertreatment may be the lack of evidence for the safety and effectivity of antidepressant medication, the reluctance of patients to adhere to antidepressant medication, the lack of mental healthcare provision in somatic healthcare environments and end-stage renal disease (ESRD) related physical limitations that complicate face-to-face psychotherapy. Guided Internet-based self-help treatment has demonstrated to be effective for depressive symptoms in other chronic patient populations and may overcome these barriers. The aim of this study is to investigate the (cost) effectiveness of a guided Internet-based self-help intervention for symptoms of depression in dialysis patients.

**Methods:**

This study is a cluster randomized controlled trial (RCT) that investigates the effectiveness of a 5-week Internet-based self-help Problem Solving Therapy (PST) for depressive symptoms in dialysis patients. Depressive symptoms will be measured using the Beck Depression Inventory – second edition (BDI-II), with a cut-off score of ≥10. We aim to include 206 dialysis patients with depressive symptoms who will be cluster randomized to the intervention or the Care as Usual (CAU) control group. Secondary outcomes will include anxiety symptoms, quality of life, economic costs and clinical outcomes, such as inflammatory factors and hair cortisol levels. Assessments will take place at baseline (T0), 2 weeks after intervention (T1) and 6 months (T2), 12 months (T3) and 18 months (T4) after intervention. The control group will be measured at the same time points. Analysis will be based on the intention-to-treat principle. Mixed models will be used to assess the changes within each condition between pre-treatment and post-treatment.

**Discussion:**

If demonstrated to be (cost) effective, Internet-based PST will offer new possibilities to treat dialysis patients with depressive symptoms and to improve their quality of care.

**Trial registration:**

Dutch Trial Register: Trial NL6648 (NTR6834) (prospectively registered 13th November 2017).

## Background

Only in a minority of dialysis patients, depressive symptoms are diagnosed and treated [1]. However, depressive symptoms are highly prevalent in this population and are associated with adverse clinical outcomes [2]. These symptoms are a major burden to the individual dialysis patient causing decreased quality of life and are associated with decreased adherence to dialysis prescription and lifestyle advice, increased hospitalization and decreased survival [1, 3, 4]. Furthermore, the social and economic costs related to depression in the dialysis population are substantial. The effect of depressive symptoms on the increase of health care costs seem to be independent of other factors, such as comorbidities, dialysis aspects and demographic variables [1, 4].

Depressive symptoms and its impairments do not remit spontaneously if left untreated [5, 6]. Underlying factors for under-treatment of depressive symptoms with medication are a lack of evidence for the safety and effectivity of antidepressant medication in dialysis patients [7, 8] and the reluctance of patients to adhere to antidepressant medication [9, 10]. Psychotherapy could be a safe alternative, with promising results in the few published trials in the end-stage renal disease (ESRD) population [11–13]. However, barriers to receive psychotherapy are the lack of mental healthcare provision in somatic healthcare environments and ESRD related physical limitations such as fatigue and other physical impairments that may reduce the ability of patients to attend face-to-face psychotherapy.

Guided online self-help cognitive behavioural treatment tailored to dialysis patients may be a promising tool for treatment of depression in this population. A guided cognitive-behavioural internet-based self-help intervention can overcome various barriers with respect to face-to-face interventions as it is easy accessible, home or dialysis-based and can be followed in one’s own limited time [14]. Self-help treatment has been proven effective in psychological distress in people with and without chronic physical health conditions [15–19]. These self-help interventions have proven equally effective in terms of reducing depressive symptoms and adherence compared to face-to-face psychological interventions, when they are guided by a therapist [20]. Feasibility trials with online self-help cognitive behavioural treatment in dialysis patients show promising results but, to the best of our knowledge, no adequately powered randomized controlled trial (RCT) has yet been performed [16, 21–24].

A commonly used brief, structured, psychological intervention adapted for use in the medical setting is Problem Solving Therapy (PST). PST is based on the assumption that depressive symptoms are caused by difficulties patients encounter in life. The goals are to teach a structured problem-solving technique to help solve the patients’ current problems and to provide a sense of mastery and self-control by providing a positive experience of problem-solving [25, 26].

Besides the lack of evidence for the effectiveness of treatment on improving depressive symptoms in dialysis patients, more insight is needed in the possible mechanisms that are involved between somatic markers and depressive symptoms in this patient population. Numerous studies have suggested a parallel inflammatory pathway between depression and ESRD via elevated levels of inflammatory cytokines [27] or a relation between hyperactivity of the hypothalamic-pituitary-adrenal (HPA) axis and depressive symptoms [28]. These pathways are not yet fully understood and are founded on associations found in observational studies. Monitoring changes in stress and inflammatory markers pre- and post-treatment may provide insight in the direction of the hypothesized causal pathways.

There are no adequately powered studies that have examined the effectiveness of guided Internet-based self-help treatment in dialysis patients. Data from applying PST in other medical settings and from smaller feasibility trials in dialysis patients show promising results in improving depressive symptoms. We hypothesize that this intervention will lead to lower depressive symptoms, is better accessible and can be offered at low costs [29, 30]. The aims of this RCT are therefore multiple. First, we will evaluate clinical and cost-effectiveness of a guided internet-based self-help PST for dialysis-patients on the primary outcome measure of depressive symptoms. Secondly, we will examine the effect on the secondary outcome measures anxiety and quality of live. And thirdly, we will examine biochemical mechanisms of depression by investigating changes in inflammatory parameters and hair cortisol levels pre- and post-treatment.

## Methods

### Study design

This study is a multicentre, cluster RCT with an active intervention arm and a Care as Usual (CAU) control arm. The intervention is a guided 5-week Internet-based self-help PST treatment, offered on tablet-computers during dialysis sessions. The internet-based treatment is based on face-to-face PST and is guided by a therapist, who gives individual feedback to the patients via the online portal. Eligible and consenting patients will be assessed at baseline (T0), within 2 weeks after the intervention (T1), at 6 months (T2), at 12 months (T3) and at 18 months (T4) after intervention. The control group will be assessed at the same time points. Data will be collected by self-reported questionnaires during dialysis sessions. In parallel we will conduct an economic evaluation in order to assess cost-effectiveness and monitor changes in stress and inflammatory markers pre- and post-treatment to examine biochemical mechanisms of depression.

**Fig. 1:**
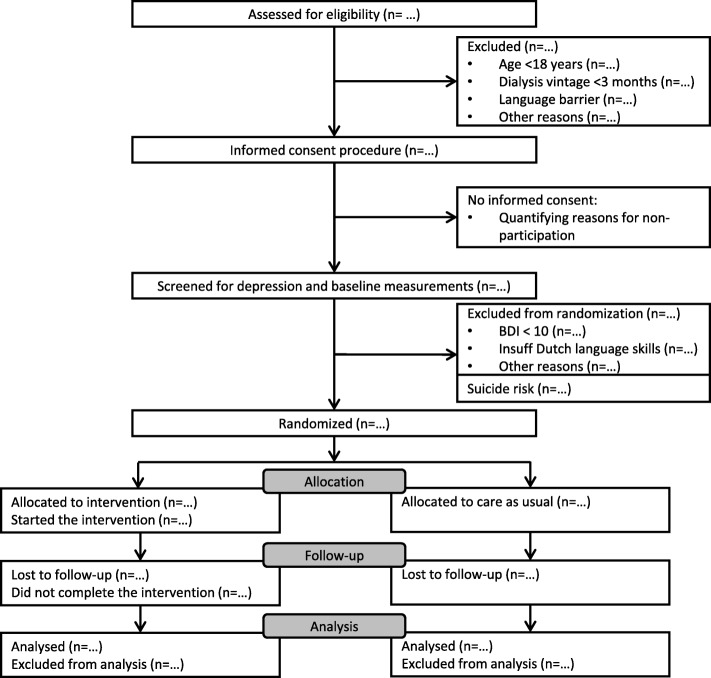
SPIRIT flow diagram

The study protocol, information brochure and informed consent were approved by the Medical Ethics Committee of MEC-U, Nieuwegein, the Netherlands (registration number: NL58520.100.17). Protocol modifications will be reported to and approved by the Medical Ethnics Committee before implementation in the trial. Written informed consent is obtained from all participants. Fig. 1 displays the flow diagram of the study design. This protocol is written in accordance with the SPIRIT guidelines.

### Recruitment

Dialysis patients will be recruited from 18 participating dialysis centres in 10 cities in the Netherlands. A list of study sites can be found in Additional file 1. We will approach and inform all eligible dialysis patients in the participating centres about the study in close collaboration with all stakeholders, such as nephrologists, nurses and social workers. The treating nephrologist will inform the patient on the trial and introduces the research assistant in the dialysis centre. If patients are willing to participate, written informed consent will be obtained by the research assistant. The attending nephrologist will be informed about participation. After giving consent, patients will be requested to complete a self-assessment questionnaire (T0) on depressive symptoms, anxiety symptoms, quality of life, dialysis symptoms and several socio-demographic questions. Blood and hair samples will be taken and stored for analysis of inflammatory parameters and cortisol levels.

### Trial inclusion criteria

Chronic, adult dialysis patients, defined as being (i) 18 years or older and (ii) > 90 days on dialysis treatment, who are (iii) able to fill in a questionnaire in Dutch and have (iii) a depressive symptoms score of 10 or higher on the Beck Depression Inventory – second edition (BDI-II) [31], will be randomized to the intervention or control arm. This cut-off value of 10 showed promising results in earlier feasibility trials [21, 23].

### Observational cohort inclusion criteria

Chronic, adult dialysis patients who are excluded from the randomization because of a low score on the BDI-II or patients who have insufficient Dutch language skills, are offered to participate in a parallel observational cohort study. Questionnaires will also be available in Arabic, English and Turkish. In this manner we will gain information on the excluded patients and thus the generalizability of the results, which could aid in the implementation in clinical practice.

### Exclusion criteria

Patients will be excluded if they are actively suicidal. If patients report suicidal ideations on item 9 of the BDI-II “suicidal thoughts and wishes” by scoring ‘2’ (“I would like to kill myself”) or ‘3’ (“I would kill myself if I had the chance”), suicide risk will be further assessed by a study doctor under supervision of a psychiatrist. If the patient is actively suicidal, the patient will be excluded from the study and the attending nephrologist will be informed and advised to refer the patients for adequate safety and treatment.

### Randomization

Cluster randomisation will be applied to reduce possible contamination between both arms of the trial. Participants will be cluster-randomized in a 1:1 allocation to the intervention or CAU after baseline measurements (T0). Clusters will be based on the shift of the dialysis session per dialysis centre. The average dialysis centre has 4 major shifts: Monday morning, Monday afternoon, Tuesday morning and Tuesday afternoon. A total of 72 clusters of average 3 patients in 18 participating dialysis centres will be present in our study. Clusters will be randomized using stratified blocks per participating dialysis centre.

Randomization will be performed and registered by an automated computer software programme to ensure independent allocation. Baseline measurements will be completed prior to randomization. The coordinating researcher assigns the intervention to participants. Although the treating nephrologist will not be actively informed about the depression score and allocation, we do not consider them blinded as patients and investigators will not be blinded. Outcome assessors are blinded and data analysts will be blinded until primary analysis will be performed.

### Intervention

The internet-based self-help intervention examined in this study is an online psychotherapy, based on PST principles [26]. PST focusses on developing coping skills and is concentrated on practical problems which people face in their daily lives. An existing evidence based internet version of PST, which has proven to be effective in similar somatically ill patient populations, [30], was adjusted for use in the dialysis population, while conserving the intent of the original PST-based intervention. Modifications concerned additional information about dialysis treatment and its psychosocial consequences, real-life examples from dialysis patient focus groups and transforming the written information into easily understandable animations. The text and animations were rewritten to comply with a ‘B1’ language reference level [32].

The intervention consists of five modules with explanatory text, animations, figures and exercises and is called ‘Worry Less for Dialysis Patients’ (in Dutch: “Minder Zorgen voor Dialyse Patiënten”). Patients are requested to complete 1 session each week and to finish the module within 6 weeks. However, there will be a possibility to extend this period up to 10 weeks, which will be documented. Patients are offered to complete the sessions on a tablet computer provided by this trial during dialysis sessions, but if preferred it can also be done from home on a private tablet or computer. If patients are unfamiliar with tablet computers, they are offered the opportunity to receive a printed booklet of the intervention. If patients have problems with the use of the tablet due to unfamiliarity, physical limitations, shunt use in the dominant arm of Dutch writing problems, they will be supported by a member of the research team, which will be documented. Supported care within the module is provided by a trained therapist and consists of weekly online feedback on their assignments. Patients have the possibility to request their therapist for additional support via the website. Treatment non-adherence and drop-outs will be discussed with the patients. Reasons for not completing the modules and patient satisfaction will be obtained via a short evaluation questionnaire. If a patient expresses suicidal ideations in the assignments of the intervention, suicide risk will be assessed over telephone by the supporting therapist under supervision of a psychiatrist. If the patient is actively suicidal, the patient will be excluded from the study and the attending nephrologist will be informed and advised to refer the patient for adequate safety and treatment.

### Control

Patients randomized to the control group do not receive Internet-based PST during standard haemodialysis treatment. Both patients in the intervention and in the control group are free to accept any medical or psychological intervention during the study (CAU). The received mental healthcare will be monitored through electronic patient records and self-reported healthcare utilization.

### Feasibility

In 2016 we conducted a feasibility test with 15 patients from different age categories in the dialysis clinic of the OLVG-West hospital in Amsterdam. Every patient was given a tablet computer with one of the modules of the PST intervention. Patients evaluated the course with a grade of 7 out of 10. The instruction and lay-out was clear for most of the patients, respectively 75 and 90%. The introduction and several explanatory texts were adjusted according to suggestions from participants in the feasibility test and patient focus groups.

Initially we used BDI > 13 as inclusion criteria. Due to new available research in feasibility trials we amended the protocol to BDI > 10 which has been approved by the Medical Ethics Committee MEC-U [21, 23].

### Outcome measures

#### Patient characteristics

Demographic self-reported items in the questionnaire will include postal code, marital status, number of children, education, profession, ethnicity, payed labour hours, smoking, alcohol usage and psychiatric disorders in the family. Data extracted from patient files will include gender, age, dialysis vintage, vascular entrance, registration on transplantation list, somatic and psychiatric comorbidities according to Davies Comorbidity Index, body mass index (BMI), primary cause of kidney failure, medication, routine laboratory measurements and change in dialysis modality. Psychotherapy and medication usage will be registered between follow-ups. The self-reported measurements will be conducted with printed self-reported questionnaires handed out during the dialysis session of the participants. Table 1 describes the measures used at each assessment point.

**Table 1:** Summary of measures

Measure	T0: baseline	T1: 2 weeks posttreatment	T2: 6 months posttreatment	T3-T4: 12–18 months posttreatment
Self-report measures				
Demographics	x			
BDI-II	x	x	x	x
BAI	x	x	x	x
QIDS-SR16	x	x	x	x
SF-12	x	x	x	x
EQ-5D	x	x	x	x
DSI	x	x	x	x
Short Tic-P	x	x	x	x
Hair questionnaire	x	x	x	
Evaluation of intervention		x		
Data extraction from patients files	x	x	x	x
Biochemical parameters	x	x	x	x
Hair cortisol	x	x	x	

#### Primary outcomes

Depressive symptoms are assessed using a self-questionnaire, the BDI-II [33, 34]. Respondents are asked to rate how much each of these symptoms bothered them in the past week, on a scale ranging from 0 (not at all) tot 3 (severely). The total score has a minimum of 0 and a maximum of 63. Treatment response will be based on the change in depressive symptoms defined by a change in the sum score of the BDI-II. The BDI-II has been validated and extensively used in the dialysis setting [2, 31, 35]. Furthermore, the Quick Inventory of Depressive Symptomatology (QIDS-SR16) self-report questionnaire will be used to assess the specific depressive symptoms, its severity and its symptom domains [36].

#### Secondary outcomes

Secondary outcome assessment based on self-report will include measures of anxiety, quality of life, health care utilization and the prevalence, severity and impact of symptoms in dialysis patients. Anxiety will be assessed with the Beck Anxiety Inventory (BAI) [37]. Quality of life will be measured using the Short Form-12 (SF-12). The 12-item Short Form Health Survey was developed for patients with chronic conditions. The SF-12 has been validated and frequently used in the dialysis patient groups [38]. Prevalence, severity and impact of symptoms of dialysis will be assessed with the Dialysis Symptom Index (DSI) [39]. Clinical outcomes include mortality and hospitalization. Mortality is measured using the European Renal Association – European Dialysis and Transplant Association (ERA-EDTA) coding system to make differences between cardiovascular and non-cardiovascular mortality. Hospitalization is defined as the number and reason of hospital admissions from baseline till end of the study period.

### Economic evaluation

A validated health-related quality of life instrument will be used to assess quality-adjusted life years (QALY) health gains. For this purpose we will use the Dutch version of the 5 level EuroQol five dimension scale (EQ-5D), a generic quality of life instrument which comprises of five domains: mobility, self-care, usual activities, pain/discomfort and anxiety/depression. The EQ-5D index is obtained by applying predetermined tariffs (utility weights) to the five domains. The Dutch tariffs of the EQ-5D will be used for computing the QALYs [40]. This index provides a societal-based global quantification of the patient’s health status. Furthermore, the EQ-5D will be compared with the SF-12 health related quality of health questionnaire, which is used often in patients on chronic dialysis therapy. Healthcare usage will be measured using part one of the Tic-P self-report together with data from patient files [41].

### Inflammatory factors and cortisol

Besides data on biochemical parameters extracted from patient files, blood samples will be taken to measure cytokines interleukin 1-beta (IL-1B), IL-6, IL-10, high sensitivity C-reactive protein (Hs-CRP) and tumour necrosis factor alpha (TNFa). Peripheral blood before dialysis will be collected in anticoagulant-free EDTA and serum tubes. All samples will be immediately centrifuged at 1200 g for 10 min and stored in aliquots at − 80 °C until analysis. Hair samples will be taken from the back of the head as close as possible to the scalp to measure mean cortisol concentrations from the past 3 months [42].

### Data management and monitoring

Patient flow in the participating centres will be organised using a secured tailor-made Access database. The decoding list will be kept in the Investigator Site File in a secured place in the dialysis department. Data-entry will be coded and entered in Castor [43]. Range limitations will be build into Castor to prevent misclassification and measurement error. Research assistants will be trained in data collection and data entry to enhance data quality.

The risk of this trial is classified as ‘negligible’ and a study specific monitoring plan is created in close corporation with the data monitoring committee which includes double data entry checks. If deemed necessary by the monitoring committee, monitoring can be intensified. No adverse events are to be expected specifically related to this intervention. Actively suicidal participants will be excluded from the trial as described above.

### Sample size

The power calculation is based on the comparison between T1 to T0 between the intervention and the control group. We took the conservative small to medium effect size (Cohen’s d = 0.4) on the primary outcome measure, while using a power 0.80, with alpha set on .05. Therefore, a total set of *N* = 99 patients is required in each arm. The design effect of cluster-randomization is estimated to be 1.04. After adjustment for cluster randomization, sample size is calculated to be *N* = 206 in total (103 per arm).

### Statistical analysis

We will quantify the flow of participants through the study using frequencies and percentages in accordance with the SPIRIT flow diagram shown in Fig. 1. Missing data will be reported and discussed in the manuscripts on the trial. Depending on the type of missing data, multiple imputation will be used to handle missing data. Descriptive statistics will be used to describe non-consent, treatment adherence and completion, drop-out and exclusion. The main analysis to assess the effectiveness of the intervention will test differences in the change in depression scores pre-and post-treatment between the intervention and control arms. Analysis will be done per intention to treat principle. Differences in change in depressive symptoms and other continuous secondary outcomes between intervention and control will be assessed using mixed models with the respective clusters, centres and baseline scores as covariates. The analyst will be blinded to the treatment group allocation. Treatment effect over time will be tested by adding a group*time interaction term into the model. Regression models will be used to explore (biochemical) mechanisms. Multivariable adjustment will be done deliberately within the causal pathway in order to explain potential mechanisms.

### Economic analysis

The economic evaluation will be conducted in two ways. First we will conduct a cost-effectiveness analysis (CEA) with treatment response as the clinical outcome of interest. Second, we will conduct a Cost-Utility Analysis (CUA) using QALYs as a generic measure of health gains. Both CEA and CUA will be conducted from both the societal perspective (including indirect costs) and the health care perspective (direct healthcare costs). Analysis will be conducted using an intention to treat principle.

The Incremental Cost-Effectiveness Ratio (ICER) will be computed to obtain costs per treatment response and the costs per QALY gained. For decision-making purposes, the ICER acceptability curve will be plotted for various Willingness-To-Pay (WTP) ceilings, which helps to making judgements whether the intervention offers good value for money relative to CAU or no treatment.

### Trial status

Enrolment for the trial started in January 2018. At the moment of submission of this manuscript, recruitment of participants is still open.

### Dissemination policy

Trial results will be published as manuscripts in international peer reviewed journals and information bulletins to participants and personnel of participating dialysis centres. In close corporation with the Dutch Kidney Patient organisation we will disseminate the trial results among dialysis patients throughout the Netherlands. No professional writers will be used during the writing of the manuscripts. Authorship eligibility guidelines according to the international committee of medical journal editors (ICMJE) will be applied to all submitted manuscripts.

## Discussion

There is a need for adequately powered RCT’s to assess the effectiveness of treatment options for depressive symptoms in dialysis patients, as evidence is currently lacking. In order to conduct a high-quality trial, a multidisciplinary team with various experts related to dialysis and depression was involved in the development of the intervention and the study design. This trial may provide insights on the effectiveness, feasibility and applicability of Internet-based self-help PST in the dialysis population.

This study has several limitations. First, the intervention may not be applicable for patients with cognitive impairment, illiteracy, other cultural background or insufficient Dutch language skills. Second, Internet-based treatment on tablet-computers might not be suitable for dialysis patients who are not familiar with the Internet or tablet-use, or who suffer from vascular disease related problems such as impaired vision or polyneuropathy of their hands. Both of these problems will be solved by offering assistance by the study team during the intervention which will be documented, furthermore patients can be provided with a printed version of the intervention. Third, drop-out rates are high for internet-based self-help interventions in other populations [29, 30], to cope with this problem we embed the intervention in routine care in the dialysis departments during dialysis treatment with frequent face-to-face interaction with the participants, which will make it easier to prevent early termination. Furthermore, we aim to provide insight in the reasons for termination or non-participation in this study.

If demonstrated to be (cost) effective, Internet-based PST offers new possibilities to treat many dialysis patients with symptoms of depression and to improve the quality of care. This RCT is aimed at contributing to better recognition and adequate treatment of symptoms of depression in dialysis patients in the future.

## Supplementary information

**Additional file 1.** List of participating centres. Table of participating dialysis centres, corresponding cities and local investigators

## Data Availability

The final trial data set will be available only to the data analysts and not to the principal investigator, other members of the study team or local investigators. In the research contract signed by all participating centres is stated that OLVG will be owner of the final trial data set. The datasets generated and/or analysed during the current study are not publicly available due to privacy reasons but will be available upon reasonable request from the principal investigator Dr. C.E.H. Siegert (e-mail: c.siegert@olvg.nl).

## Abbreviations

BAI: Beck Anxiety Inventory
BDI-II: Beck Depression Inventory – second edition
BMI: Body Mass Index
CEA: Cost-Effectiveness Analysis
CUA: Cost-Utility Analysis
DSI: Dialysis Symptom Index
EQ-5D: 5 level EuroQol five dimension scale
ERA-EDTA: European Renal Association – European Dialysis and Transplant Association
ESRD: End Stage Renal Disease
HPA: Hypothalamic-pituitary-adrenal
Hs-CRP: High sensitivity C-reactive protein
ICER: Incremental Cost-Effectiveness Ratio
IL-1B: Interleukin 1-beta
PST: problem solving therapy
QALY’s: Quality-adjusted life years
QIDS-SR16: Quick Inventory of Depressive Symptomatology
RCT: Randomized Controlled Trial
SF-12: Short Form 12
TNFa: Tumour necrosis factor alpha
WTP: Willingness-To-Pay
