# Supplementary material for: The (cost) effectiveness of guided internet-based self-help CBT for dialysis patients with symptoms of depression: study protocol of a randomised controlled trial
Source: BMC Psychiatry. 2019 Nov 27;19:372. doi: 10.1186/s12888-019-2363-5 (PMC6881986; doi:10.1186/s12888-019-2363-5)
Supplement: Supplementary file 1 — Additional file 1. List of participating centres. Table of participating dialysis centres, corresponding cities and local investigators [file 12888_2019_2363_MOESM1_ESM.docx]

**Supplement 1: List of participating centres**

| **Dialysis centres** | **City** | **Local investigators*** |
| --- | --- | --- |
| OLVG West  OLVG Oost | Amsterdam  Amsterdam | C.E.H. Siegert, internist-nephrologist |
| Amsterdam University Medical Centres, location VUmc  Diapriva  Niercentrum aan de Amstel | Amsterdam  Amsterdam  Amstelveen | F. van Ittersum, internist-nephrologist |
| Haaglanden Medisch Centrum, Westeinde  Haaglanden Medisch Centrum, Antoniushove | The Hague  The Hague | P. Chandie-Shaw, internist-nephrologist |
| HagaZiekenhuis Leyweg  HagaZiekenhuis Sportlaan  HagaZiekenhuis DialyseCentrum Zoetermeer | The Hague  The Hague  Zoetermeer | L.J. Vleming, internist-nephrologist |
| Jeroen Bosch Ziekenhuis | Den Bosch | E. Hoogeveen, internist-nephrologist |
| St. Antonius Ziekenhuis  St. Antonius Dialysecentrum Tiel | Nieuwegein  Tiel | W.J.W. Bos, internist-nephrologist |
| Maasstad Ziekenhuis | Rotterdam | M. Dekker-de Bie, internist-nephrologist |
| Franciscus Gasthuis  Franciscus Vlietland | Rotterdam  Schiedam | M. Westerman, internist-nephrologist |
| Tergooi Ziekenhuis  Dialysecentrum ‘t Gooi | Hilversum  Hilversum | M. Schouten,  internist-nephrologist |

Table of participating dialysis centres, corresponding cities and local investigators

*Local investigators are responsible for the coordination of the trial in the corresponding dialysis centres. Research assistants will work in close cooperation with the local investigators to implement this study and improve uniform inclusion and data collection.
